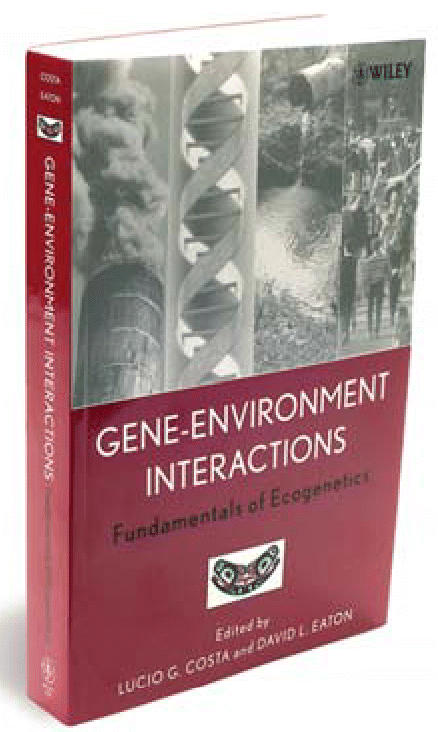# Gene–Environment Interactions: Fundamentals of Ecogenetics

**Published:** 2006-06

**Authors:** Ronald E. Cannon

**Affiliations:** *Ronald E. Cannon is a molecular biologist for the National Center for Toxicogenomics (NCT) at NIEHS, and holds adjunct faculty positions at North Carolina State University and the University of North Carolina, Chapel Hill. His laboratory has helped develop transgenic mouse models as adjuncts to the two-year mouse bioassay used to identify chemical carcinogens. His basic laboratory research program in the Cancer Group of NCT focuses on the modulation of* ras*-mediated tumors in mice.*

Edited by Lucio G. Costa and David L. Eaton

Hoboken, NJ:John Wiley & Sons, 2006. 450 pp. ISBN: 0-471-46781-2. $110.

Specific hereditary susceptibility has now evolved into the field of ecogenetics. If you have ever pondered why an individual is at greater risk for a specific disease, or sensitive to a specific drug, then *Gene–Environment Interactions* is for you. Ecogenetics has emerged in large part because of the advancements in technology and informatics in the past 10 years. These technologies have provided a better understanding of the enormous influence the environment has on gene expression. This textbook gives the reader the first extensive look at how environmental factors in concert with genetic variation modulate the various biologic components to bring about increased risk from chemicals, change drug metabolism, and predispose humans to various diseases.

The book is well written and organized, containing 25 chapters arranged into four distinct sections, which cover ecogenetics, polymorphisms, specific medical problems, and social, ethical, and legal issues. The first section provides a historical overview of ecogenetics and introduces the reader to the epidemiologic and statistical approaches in addition to the biologic tools and techniques relevant to understanding and analyzing the data. The section provides the reader with the fundamentals of the present-day molecular techniques used to acquire and analyze multi-gene expression and specific gene sequences. These techniques are well described and illustrated.

The latter part of the first section describes the statistical and epidemiologic approaches used to evaluate the influence the environment has on human disease risk. It more than adequately provides the basic concepts in statistics used in human and population genetics. The chapters dedicated to epidemiologic discussions deal with issues related to study designs and methodology design considerations to control for a specific bias.

The second section tackles the monumental task of phase I and phase II biotransformation: introducing cytochrome P450 (CYP) enzymes and describing the biologic effects and implications of CYP polymorphisms to human health and risk assessment. In this section the contributors also discuss the toxic effects of chemicals on individuals with genetic polymorphisms that compromise DNA repair machinery, rendering individuals more susceptible to genotoxic agents. The latter part of the section uses selected examples of genetic polymorphism in genes encoding receptors and ion channels to illustrate biochemical, pharmacologic, and clinical consequences.

The third section is by far the largest section of the book, containing nine chapters that examine gene–environment interactions as a function of the outcome on human health. The authors examine lung and gastrointestinal cancers, neurodegenerative diseases, cardiovascular diseases, diabetes, and infectious diseases, providing discussion on the environmental risk factors and candidate genes as well as the pathogenesis of each. The section closes with two chapters that discuss the importance of genetic determinants to nutrition and drug addiction/abuse.

The final section deals with the social implications of ecogenetic data as well as the applications of these data to risk assessment and risk management, public health, and regulatory policies. The ethical and regulatory issues entail many conceptually new and unresolved aspects of acquiring, protecting, and using one’s genetic data to determine health risk in today's world. Issues considered include susceptibility testing in the workplace, confidentiality, and, significantly, genetic discrimination. The book clearly conveys that the new frontier of ecogenetics is upon us: in health, at work, and in our everyday personal lives. Most important, it conveys that many important social, ethical, and regulatory decisions are still before us.

*Gene–Environment Interactions* is a “first of its kind” textbook dedicated to understanding the fundamentals of ecogenetics. The text is well referenced, containing an extensive bibliography. The subject matter is presented clearly with well-constructed illustrations that capture the concepts presented. The book is a must-have for all toxicology and pharmacology students as well as a great resource for researchers and physicians.

## Figures and Tables

**Figure:**